# Inhalation technique-related errors after education among asthma and COPD patients using different types of inhalers – systematic review and meta-analysis

**DOI:** 10.1038/s41533-025-00422-0

**Published:** 2025-03-18

**Authors:** Monika Marko, Rafał Pawliczak

**Affiliations:** Department of Immunopathology, Faculty of Medicine, Division of Biomedical Science, Medical University of Lodz, Lodz, Poland

**Keywords:** Health care, Diseases, Asthma, Chronic obstructive pulmonary disease

## Abstract

In asthma and chronic obstructive pulmonary disease (COPD) incorrect use of inhalers is still common. The aim of the study was to detect whether education improves inhalation skills and whether the type of education influenced the educational effect depending on the device. A systematic review and meta-analysis for errors during inhalation before and after education was performed. The selected data allowed for education assessment of dry powder (DPIs) and pressurised metered dose (pMDI/MDIs) inhalers in a meta-analysis. Education reduced critical errors (risk ratio [RR], 0.28, 95% confidence interval [CI]: 0.17, 0.47, P < 0.00001) and any incorrect use events for DPI (RR = 0.38, 95% CI: 0.21, 0.70, P = 0.002) and pMDI/MDIs, (RR = 0.16, 95% CI: 0.11, 0.23, P < 0.00001). Education improves patient’s inhalation skills regardless of the device. The educational effect for pMDI/MDIs depends on the type of educational approach which has not been demonstrated for DPIs.

## Introduction

The chronic respiratory diseases with the highest incidence include asthma and chronic obstructive pulmonary disease (COPD). Although these diseases differ mainly in pathogenesis, disease progression, prognosis and treatment options, they have similar symptoms, such as cough, shortness of breath and sputum^1^. However, the common denominator for these diseases is that the mainstay of their treatment are inhaled medications^2,3^. According to the Global Initiative for Asthma (GINA) 2024 Guidelines^3^ patients still need education and training to use inhaler devices effectively. Also, the Global Initiative for Chronic Obstructive Lung Disease 2024 (GOLD)^2^ recommends providing education on proper inhalation techniques because it is crucial to obtain optimal results from inhaled therapy. This, in turn, is key to managing asthma and COPD. It is known that education on the correct inhalation technique significantly improves patients’ inhalation skills^4^. A wide range of inhaler devices are available, including metered dose inhalers (MDIs) with or without spacer and breath-actuated MDIs (BAIs), dry powder inhalers (DPIs), soft mist inhalers (SMIs) and nebulizers^2,5^. However, it is not entirely clear whether the type of inhaler used influences the satisfactory result (for the purpose of this study defined as achieving correct inhalation technique) after the education. Also, no educational method is recognized as the standard of care^6^. It is also still not defined which type of education is the most effective. The most common methods of inhalation technique education are brief intervention (BI)^7,8^, and “teach-to-goal” (TTG)^9^. BI is based on giving the patients verbal instructions without demonstrations^8^. On the other hand, TTG is an educational strategy consisting of multiple sessions during which patients learn self-care skills until the learning goals are achieved^9,10^. This approach can be divided into virtual^11^, verbal, in-person^12^ or video TTG method^10^. The “teach-back” approach, which involves the patients demonstrating and explaining what a health care professional (HCP) has taught them in their own words, is also considered highly effective^2,3,13^. This educational approach is designed to ensure that patients understand the instruction. The patient is re-educated if they are unable to explain or demonstrate the technique correctly. Education is continued until patients have mastered the correct inhalation technique^14^.

The GOLD raises the critical point that patients typically receive adequate education and follow-up on inhalation device skills in randomized controlled trials (RCTs) and may, therefore, not reflect everyday clinical practice. Consequently, it is essential to consider real-life study results^2^. It has been shown that improper inhalation technique in patients with asthma and COPD is still common in real-life and is associated with poor clinical control of these diseases^4^. The basic problem is that most patients claim to know how to use the inhalation device, but 94.2% make at least one error^15^. In addition, patients require monitoring of inhalation technique and training from an HCP when using any type of device^2,16^. This statement is also confirmed by the GINA, highlighting the additional problem that most patients need to be made aware of that they are making mistakes during inhalation and that the HCP cannot demonstrate how to use the inhaler properly^3^.

In everyday practice, an HCP must choose which inhaler to use for a given patient. It is often necessary to consider whether the prescribed device will be easy to use and, most importantly, whether the patient will use it correctly. There is no doubt that knowledge of inhalation devices, along with demonstration of the inhalation technique, helps optimize treatment outcomes^17^. Another important factor is the selection of the appropriate educational method regarding the use of the inhaler. Therefore, numerous studies have been attempting to evaluate inhalers, device mastery and various educational approaches^8,10,11,18–20^. Research examining factors influencing adherence to therapeutic recommendations and proper use of inhalers also plays an important role^21,22^. It has been shown that the correct inhalation technique is related to their knowledge of the disease process, age, gender, educational status, and training in the use of inhalation devices^23^.

In this study, a systematic review and meta-analysis was conducted to assess the occurrence of errors in the inhalation technique before and after educating patients on the use of different types of inhalers in the treatment of asthma and COPD. An attempt was made to detect whether education improves inhalation skills and to assess whether the type of education influenced the educational effect according to the device.

## Methods

### Search strategy and selection criteria

A systematic review and meta-analysis were performed according to the protocol described in PROSPERO (ID: CRD42024560342). PubMed/Medline, Embase, Web of Science, ClinicalTrials.gov, and Cochrane Central Register of Controlled Trials (CENTRAL) databases were thoroughly searched. Additionally, Google Scholar was searched for grey literature (conference proceedings, non-peer-reviewed publications, reports, datasets, patents, and whitepapers). The search was conducted from 23 June 2024 to 21 July 2024. The search strategy is presented in Table 1 according to Preferred Reporting Items for Systematic Reviews and Meta-Analyses (PRISMA)^24^. The searches were re-run before the final analysis to identify further studies and possibly include them.

**Table 1:** Search strategy.

Database	Search strategy
PubMed/Medline (search: 01.07.2024; re-run search^a^: 22.07.2024)	((inhaler) OR (inhalers) OR (inhalation)) AND ((incorrect technique) OR (errors) OR (misuse)) AND ((education) OR (training) OR (demonstration))
Cochrane Central Register of Controlled Trials (CENTER) (search: 15.07.2024; re-run search^a^: 23.07.2024)	((inhaler) OR (inhalers) OR (inhalation)) AND ((incorrect technique) OR (errors) OR (misuse)) AND ((education) OR (training) OR (demonstration))
Web of Science (search: 07.07.2024 re-run search^a^: 24.07.2024)	inhaler or inhalers or inhalation and incorrect technique or errors or misuse and education or training or demonstration (title)
ClinicalTrials.gov (search: 15.07.2024 re-run search^a^: 25.07.2024)	inhaler and inhalers and inhalation and incorrect technique and errors and misuse and education and training and demonstration with additional search options “completed”, “with results”
Embase (search: 07.07.2024; re-run search^a^: 26.07.2024)	((inhaler) OR (inhalers) OR (inhalation)) AND ((incorrect technique) OR (errors) OR (misuse)) AND ((education) OR (training) OR (demonstration)) with additional search options: “full text”, “human”, “English Language”, “Remove MEDLINE Records”
Google Scholar (search: 21.07.2024; re-run search^a^: 27.07.2024)	(inhaler) OR (inhalers) OR (inhalation) AND (incorrect technique) OR (errors) OR (misuse) AND (education) OR (training) OR (demonstration)

^a^The searches were re-run before the final analysis to identify further studies and possibly include them.

The research question and selection criteria were formulated using the Population, Intervention, Comparison and Outcome (PICO) structure^25^. The inclusion criteria were: (1) population: patients with asthma and/or COPD, (2) intervention: education in inhalation technique, (3) comparison: effect before education, (4) outcomes: must contain section reporting patients’ outcomes (overall and/or critical errors) in inhalation technique before and after education (5). Additionally, to the obligatory PICO structure, we added type of study (T): randomized clinical trials (RCTs), non-randomized clinical trials, real-life trials and observational trials, open-label trials and prospective trials.

The following exclusion criteria were formulated: (1) review article and systematic reviews, (2) case series, (3) case report, (4) meta-analysis, (5) articles with insufficient information and data (6) articles published in languages other than English, (7) original articles where specific data and outcomes could not be extracted, (8) original articles that do not specify the type of inhaler used and (9) original articles that not specify outcomes before and after education.

### Study selection and data extraction

The first selection of studies involved reviewing articles’ abstracts and titles available in databases. The search was performed by two researchers independently at the same time. After excluding duplicates, each article that met the inclusion and exclusion criteria underwent a full-text review. Two researchers assessed whether to include or reject the study independently to reduce the potential risk of selection bias. For study selection and data extraction, two separate sheets were created for each investigator to collect information about studies and to identify multiple reports. Subsequently, the content of the sheets was compared in terms of the duration of the study and date, study identification numbers (if available), names of authors and institutions, intervention details, the number of participants and outcomes.

In the next step, data extraction from selected studies was performed by each investigator. This process was conducted with meticulous attention to detail, ensuring the accuracy and reliability of the research findings. Extracted data were then cooperatively reviewed by the researchers. In case of conflicting views on the classification of results, the researchers conducted negotiations until a consensus was reached, further ensuring the accuracy and reliability of the research. The following information was extracted from included studies: title, authors, study design, number and age of subjects, intervention and type of inhaler device, disease, type and number of error cases in inhalation technique and education in inhalation technique procedure. Additionally, selected publications were screened for missing or unclear information. Data presented as a percentage were converted to quantitative data.

### Assessment of the risk of bias and methodological quality

The methodological quality and risk of bias assessment was performed by two researchers individually by using Cochrane Review Manager 5.4 software. Included studies were assessed for seven items of criteria for judging risk of bias in the Risk of bias assessment tool: (1) random sequence generation (selection bias), (2) allocation concealment (selection bias), (3) blinding of participants and personnel (performance bias), (4) blinding of outcome assessment (detection bias), (5) incomplete outcome data (attrition bias), (6) selective reporting (reporting bias) and (7) other bias. The following classification was used: low risk of bias, unclear risk of bias, and high risk of bias. The evaluation of the studies made independently by two investigators was compared. In the event of conflicting opinions, decisions were made during a discussion session, where the allowable value of losses affecting the study results was set at 10%. Additionally, we checked whether there was a conflict of interest in the included studies due to the relationship between the results and the funding sources.

### Statistical methods

Extracted data for quantitative analysis were collected in a standardized database and analyzed using Cochrane Review Manager 5.4 software. The results comprised dichotomous data displayed as risk ratio (RR) and 95% confidence interval (CI) in each group. Because heterogeneity was suspected after including the studies in the meta-analysis, the Mantel-Haenszel test with a random effects model was used for all results. This approach was intended to minimize the risk of bias in selecting effect sizes and to optimally use extracted data from studies that differed in participant composition and clinical heterogeneity. The Cochran’s Q test and I square (I^2^) indices were used to assess heterogeneity between study results. The degree to which the effect estimates of each study were distributed around the pooled effect estimates was also visually examined using forest plots. The results of were considered statistically significant at P < 0.05.

### Additional analyses

Studies included in the meta-analysis (quantitative analysis) were additionally assessed for certainty of the evidence using the Grading of Recommendations Assessment, Development and Evaluation (GRADE) approach^26,27^. The quality evaluation of included studies considered assessment of risk of bias, inconsistency, indirectness, imprecision, and publication bias. Subsequently, studies were classified as high, moderate, low, or very low certainty using standardized GRADE terminology^28,29^. To summarize the results of the assessment, we used the Summary of Findings (SoF) table^28^.

Sensitivity analysis was performed to assess the robustness of meta-analysis considering studies with high risk of bias (detected during risk of bias assessment). For this purpose, we compared the results of the meta-analysis including studies with high risk of bias with the results after excluding them from the meta-analysis.

We could not perform funnel plot analysis to assess publication bias because, according to Cochrane recommendations^30^ for meta-analyses that do not include at least 10 studies, the power of this test is too low to distinguish real asymmetry.

## Results

### Included studies

**Fig. 1:**
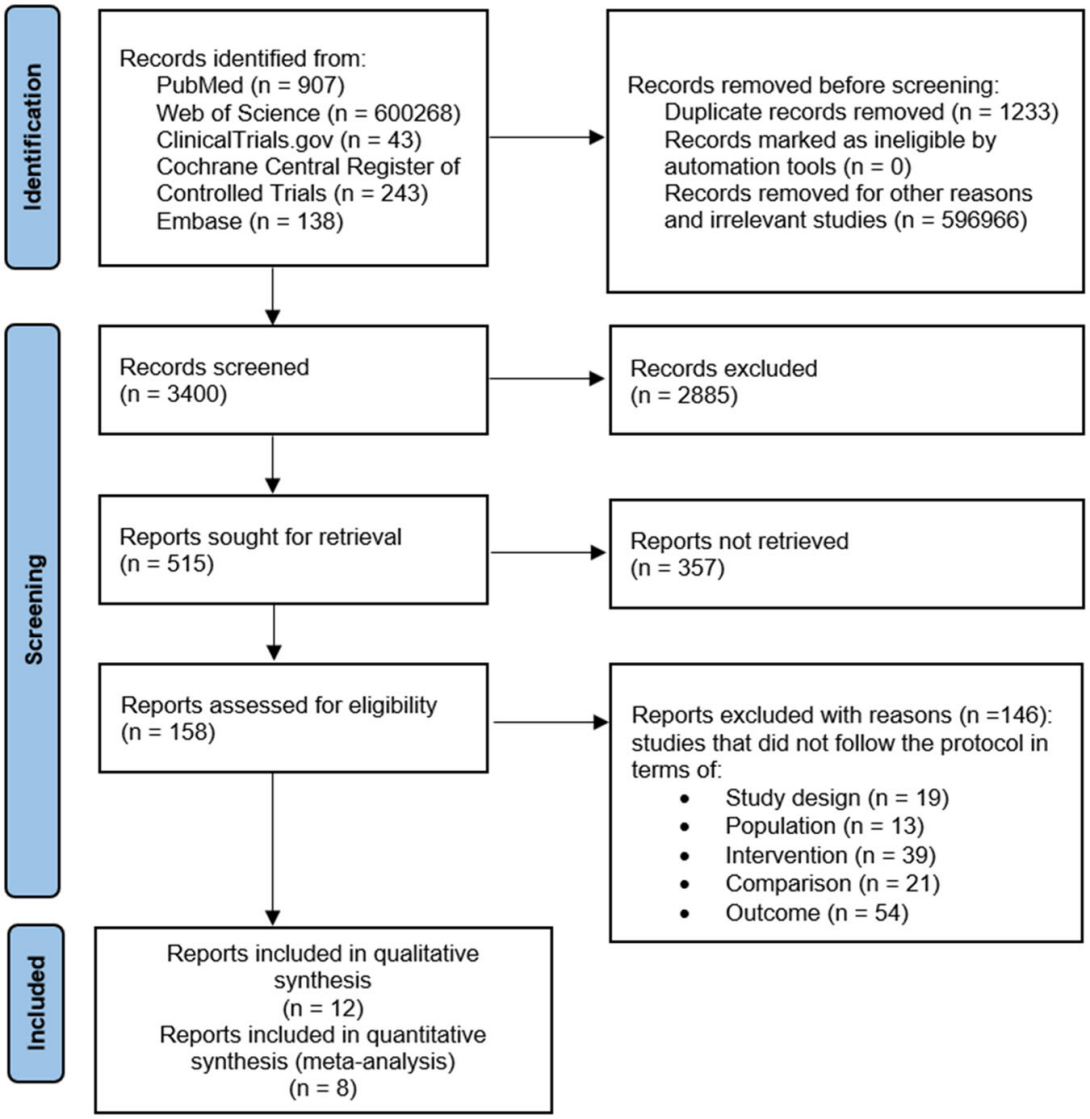
Flowchart of the screening procedure.

Figure 1^24^ shows that the records screened included 3400 related articles. After full-text screening, assessment for eligibility and quality evaluation, twelve articles meeting the inclusion criteria were included for qualitative analysis^10,17,21,31–34^. Whereas 146 reports were excluded with reason. Included studies were randomized clinical trials (RCTs), non-randomized clinical trials, real-life, observational trials, open-label trials and prospective trials. It was assumed that one analyzing group or subgroup in meta-analysis, could not include both randomized and non-randomized studies.

All the articles provided results for several inhalation devices and educational approach in inhalation technique and were conducted on asthma or COPD patients. We also included asthma-COPD overlap syndrome (ACOS) studies^31^. Studies that compared more than one educational approach or/and inhaler device and disease (asthma and/or COPD) were presented in the meta-analysis in separated parts.

Of the selected studies, all included adults or adolescents (≥15) except one, which included both adult and pediatric patients in one group (5–80)^32^. Combining these outcomes, may contribute to misleading results. Given that, the results for this group from that study were not included in the meta-analysis.

Because both educational methods and inhaler type may influence the effect of education, separate subgroups were used to avoid the risk of biased results in the meta-analysis.

Studies were grouped and included in the analysis based on data regarding device type and type of education. In addition, type of error made, and previous education (if reported by the authors) were considered. Preliminarily qualified studies were rejected if their results did not correspond to the assumed groups in the meta-analysis. Errors referred to in PICO as “overall errors” have been replaced with the term “any incorrect use events”. This change was made because when planning the research question according to the PICO, we assumed that “overall errors” are any errors, incorrect use, and incorrect inhalation techniques. By definition, an “overall error” is any non-critical error in inhaler use^35^, and using the term “overall errors” in the PICO research question may confuse the reader. Consequently, we divided the errors into two types (“critical” and “any incorrect use events”).

After quality assessment, eight studies were included for quantitative analysis^7,8,10,17,21,31–33^. Only in one study^27^ eligible for meta-analysis authors declared that they described patients who had not used the inhalers covered by the study for at least a year, so a separate group was created. Study details are shown in Table 2. Additional information regarding the characteristics of the studies included in the systematic review and meta-analysis is presented in Supplementary Table 1 and Supplementary Table 2. No literature item was included in the study at the grey literature search stage (Google Scholar), and no duplicates were found. After re-run search, no additional studies were included in the systematic review and meta-analysis.

**Table 2:** Characteristics of studies included in systematic review and meta-analysis.

Study ID	Study design	Inhaler	Sample size	Age and disease	Education in inhalation technique procedure
Ahn et al.^58^	a prospective cohort study	DPI^a^, pMDI^a^, SMI^a^	261	>40 COPD	Face to face training using the “teach-back” technique provided by the nurse
Aksu et al.^31^	Prospective study	pMDI/MDI, DPI^a^	108	≥18; asthma and/or COPD, asthma COPD overlap syndrome	1. Assessment of inhaler technique (own inhaler device). 2. Training on the use of the device. 3. Inhaler technique assessment (after 3 months).
Al-Kharouf et al.^10^	A prospective, open label, randomized controlled trial	pMDI^a^ Respimat^a^ Turbohaler, Accuhaler, Breezhaler^a^	103	45–62; asthma and/or COPD	1. Inhaler technique assessment at baseline. 3. Participants received either a standard verbal TTG education or a video-based TTG education. 2. Inhaler technique assessment at follow-up (after 3 months).
Aydemir et al.^21^	a real life, cross-sectional study and no intervention	DPI pMDI/MDI	342	15–84; asthma and/or COPD	1) Assessment of inhaler technique (own inhaler device). 2) Face to face training sessions. 3) Assessment of inhaler technique immediately after the training.
Balamurugan et al.^32^	open-label, prospective, comparativemulti- center study	pMDI, Synchrobreathe^a^	162 (COPD), 239 (asthma), 59 (inhaler-naive healthy volunteers^b^)	Asthma: 5–80^c^ COPD: 40–80; asthma and/or COPD, healthy volunteers^b^	1) Assessment of inhaler technique (patient previously used pMDI with spacer and healthy volunteers read leaflet) 2) Patients were provided training on using the pMDI. 3) Assessment of inhaler technique. 4) All the participants read the patient information leaflet for Synchrobreathe 5) Assessment of inhaler technique. 6) Training on the use of the device. 7) On day 14 patients demonstrated inhalation technique without training. 8) Assessment of inhaler technique.
Brusselle et al.^57^	multi-centre, single arm, non-interventional, phase IV study	pMDI^a^	126	Mean age: 66.2 COPD	Patients were trained by an HCP in the correct use of inhaler device using the MyPuff® app and/or the correct use leaflets.
Chrystyn et al.^34^	randomised, crossover, open-label study	Diskus^a^ Pulmoject^a^ Turbohaler^a^ (empty devices)	277 in Pulmojet-Diskus and 44 in Pulmojet-Turbohaler (naive to the study devices).	≥18; asthma and/or COPD	1) Patients reading the patient information leaflet. 2) Patients performed inhalation. 3) Patients watched a training video if serious errors potentially affecting dose delivery were recorded. 4) Patients repeated the inhalations.
Kim et al.^6^	prospective clinical study	pMDI^a^	62 (patients who were regularly using pMDI with or without spacers).	≥18 years and ≤90; COPD	1) Rapid cognitive screen. 2) Inhaler technique assessment. 3) An active one-on-one coaching was provided (based on 12-step American Thoracic Society instructions). 4) Inhaler technique assessment and scored again at follow-up visits.
Nitya et al.^17^	a prospective cross-sectional study	pMDI DPI pMDI with spacer^a^	144	>18; asthma and/or COPD	1. Assessment of inhaler technique. 2. A face to face training session. 3. Assessment of inhaler technique on the next day after training.
Press et al.^8^	Phase-II, block randomized, stratified clinical trial	pMDI/MDI^a^, DPI	50	≥18; asthma and/or COPD	1. Baseline inhaler assessment. 2. BI: single-set of verbal and written step-by-step instructions, -to-goal or TTG: BI plus repeated demonstrations of inhaler use and participant comprehension assessments (teach-back). 3. Post education assessment.
Press et al.^7^	two-site, block-stratified randomized clinical trial	Diskus and pMDI with spacer^a^	120	≥18; asthma and/or COPD	1) Assessment of inhaler technique (pre-education). 2) Participants were randomized to TTG or brief intervention. 3) Assessment of inhaler technique.
Van der Palen et al.^33^	randomised, cross-over study	Elpenhaler and Diskus (devices containing only placebo)	52 men and 61 women (naive to Diskus and Elpenhaler for at least 1 year)	≥40; asthma and/or COPD	1) Patients read the instructions of device and attempted to first inhalation. 2) Assessment of errors. 3) When mistakes were made, correct inhaler use was demonstrated. 3) Patients had to demonstrate again, and mistakes were registered (the procedure was repeated 4 times).

*DPI* dry powder inhaler, *pMDI* pressurized metered dose inhaler, *SMI* soft mist inhaler, *COPD* chronic obstructive pulmonary disease, *BI* brief intervention, *TTG* teach-to-goal, *HCP* health care professional.

^a^Results not included in the meta-analysis due to lack of reference group.

^b^Outcomes of healthy volunteers were excluded from systematic review and meta-analysis.

^c^Pediatric and adult participants in one group. These outcomes may contribute to misleading results. Outcomes were excluded from meta-analysis.

### Assessment of methodological quality

**Fig. 2:**
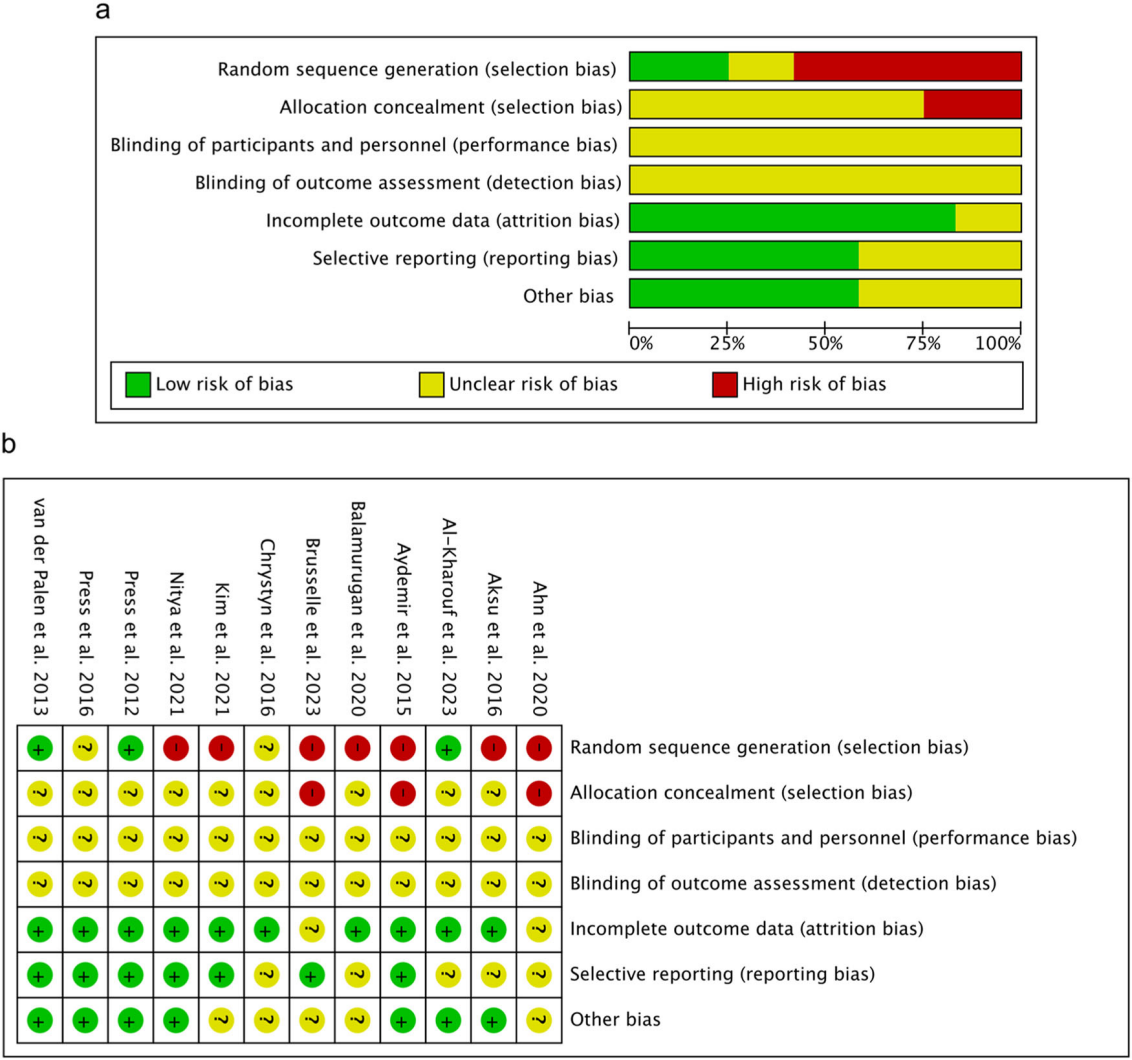
Risk of bias assessment. **a** Risk of bias summary: review authors’ judgements about each risk of bias item for each included study. **b** Risk of bias graph: review authors’ judgements about each risk of bias item presented as percentages across all included studies.

Our comprehensive evaluation process included twelve studies on the risk of bias assessment. The assessment of methodological quality revealed that all included studies have an unclear risk of performance and detection bias. For attrition bias, low risk was observed in 10 (83.33%) studies. Two studies (16.67%) showed unclear risk of attrition bias. For random sequence generation, 7 (58.33%) of the included studies have a high risk of bias, 2 (16.66%) studies showed an unclear risk, and 3 (25.00%) studies showed a low risk of this bias. For allocation concealment, 3 (25.00%) of included studies has a high risk of bias. The remaining 9 (75.00%) studies showed an unclear risk of these biases. In the case of reporting bias, low risk was detected for 7 (58.33%) and unclear risk for 5 (41.67%) of included studies. In other biases, low risk was detected for 7 (58.33%) studies, whereas unclear risk was detected for 5 (41.67%) studies. The results obtained from the methodological evaluation are shown in Fig. 2A, B. We did not detect strong evidence of publication bias after qualitative assessment of included studies.

### Critical errors

**Fig. 3:**
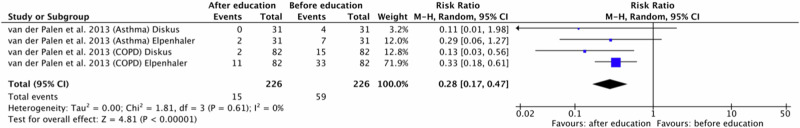
Risk ratio for critical errors in patients using DPI inhalers before and after education in form of demonstration. DPI dry powder inhaler, COPD Chronic obstructive pulmonary disease.

One original paper^27^ was eligible for meta-analysis for critical errors in the inhaler technique. The study was divided into four because the authors presented the results of asthma and COPD patients separately for two different DPI inhalers (Diskus and Elpenhaler). Polled data provided 226 patients in the experimental group (after education) and 226 patients in the comparison group (before education). In this study, demonstration was the educational approach. It can be observed that all patients, regardless of the DPI inhalation device used, improved their inhalation technique after assessed educational approach. A meta-analysis demonstrated significantly fewer critical errors occurrences in the experimental group compared to the comparison group (RR = 0.28, 95% confidence interval [CI]: [0.17, 0.47], P < 0.00001, I^2^ = 0%, P = 0.61). It should be emphasized that this analysis included participants who had not used the type of inhaler assessed for at least one year. Consequently, we can state that in the case of DPI devices, education in the form of demonstration contributes to reducing the number of critical errors. The results are shown in Fig. 3.

### Any incorrect use events

**Fig. 4:**
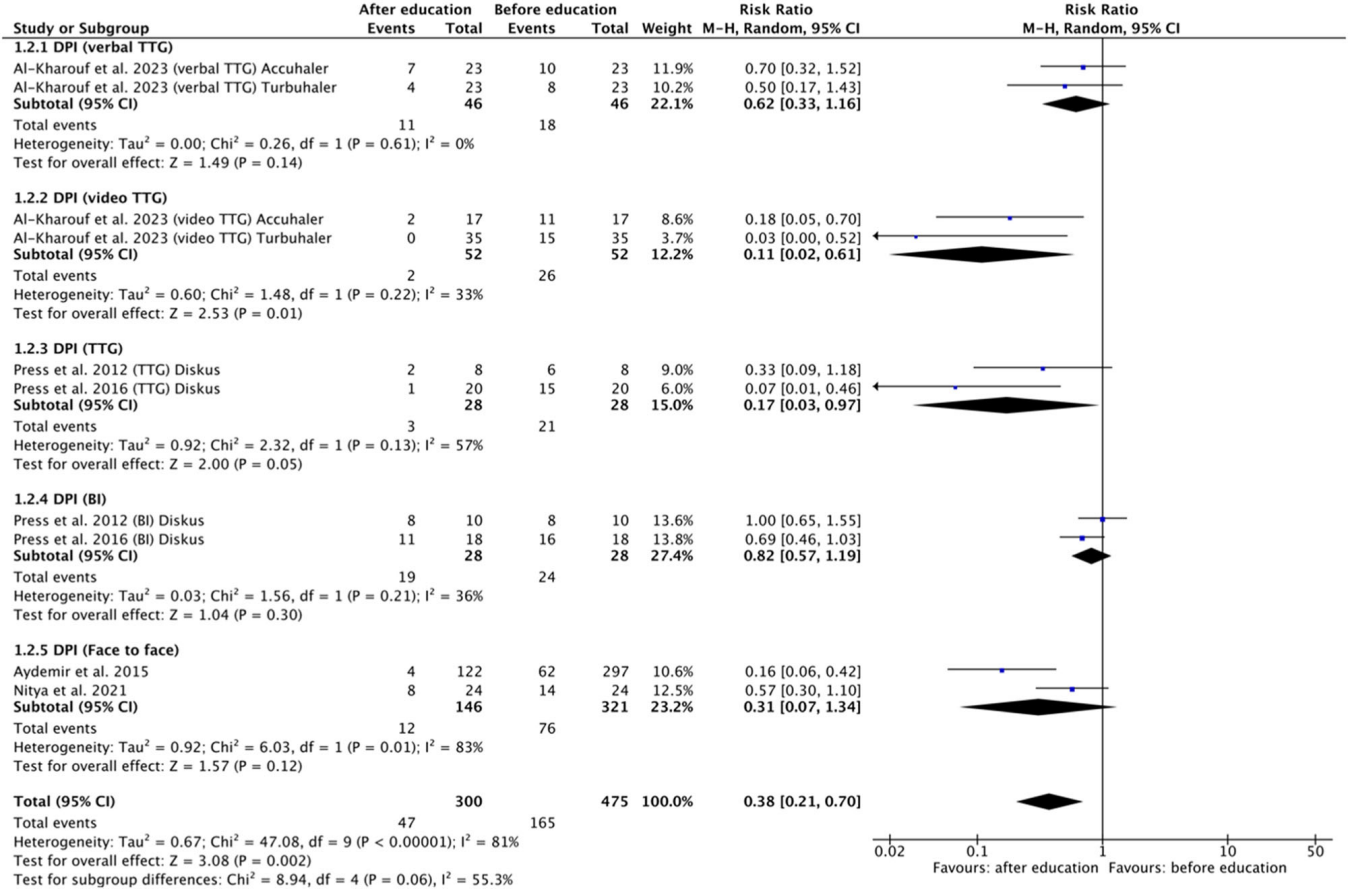
Risk ratio for any incorrect use events in patients using DPI inhalers before and after education based on the educational approach type. DPI dry powder inhaler, BI brief intervention, TTG teach-to-goal.

**Fig. 5:**
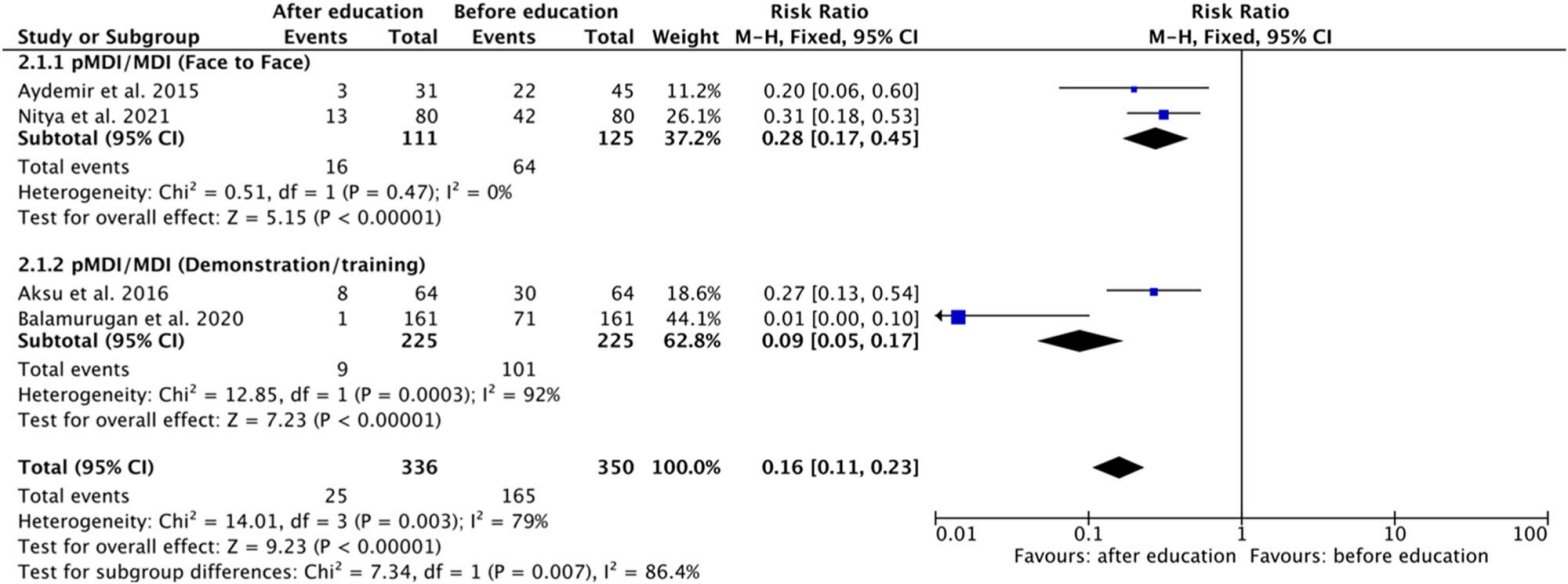
Risk ratio for any incorrect use events in patients using pMDI/MDI inhalers before and after education based on the educational approach type. Data from the pMDI/MDI studies in the meta-analysis did not include the use of a spacer. pMDI: pressurized metered dose inhaler.

For any incorrect use events in inhaler technique, we conducted two subgroup meta-analyses by educational approach. Separate forest plots were prepared for DPI and pMDI/MDI devices (Figs. 4 and 5).

For DPI five original papers^7,8,10,17,21^ were eligible for meta-analysis. One study^10^ were divided into four because the authors presented the results of different types of education: verbal TTG vs. video TTG and different DPI inhaler devices: Accuhaler and Turbuhaler. Two studies were divided into two because involved outcomes of two educational approach: TTG and BI^7,8^, both for one DPI inhaler device: Diskus. Another two studies involved face to face educational method.

Polled data provided 300 patients in the experimental group (after education) and 475 patients in the comparison group (before education). In included studies patients, regardless of the DPI inhaler device type, improved their inhalation technique after all the types of provided education. Only in one group (BI, Diskus)^8^ the number of errors after education was equal to the number of errors before education. Independent subgroup analysis based on the type of provided education, demonstrated significantly fewer errors occurrences in the experimental group compared to the comparison group for DPI and video TTG (RR = 0.11, 95% confidence interval [CI]: [0.02, 0.61], P = 0.01, I^2^ = 33%, P = 0.22).

Furthermore, the test for subgroup overall effect showed a significant improvement in inhalation skills after education for assessed inhaler devices and educational approach in case of incorrect use events (RR = 0.38, 95% confidence interval [CI]: [0.21, 0.70], P = 0.002, I^2^ = 81%, P < 0.00001).

However, the test for subgroup differences indicates that there is no statistically significant subgroup effect (p = 0.06, I^2^ = 55.3%). Accordingly, we cannot state that the effect of education depends on the type of educational approach. The results are shown in Fig. 4.

For pMDI/MDI four original papers^17,21,31,32^ were eligible for meta-analysis. Studies involved outcomes of two educational approach: face to Face method^17,21^ and demonstration/training^31,32^. Polled data provided 336 patients in the experimental group (after education) and 350 patients in the comparison group (before education). A subgroup meta-analysis showed that patients improved their inhalation technique after the education. In independent subgroup analysis based on the type of provided education, a meta-analysis demonstrated significantly fewer incorrect use events in the experimental group compared to the comparison group for pMDI/MDI and face to face method (RR = 0.28, 95% confidence interval [CI]: [0.17, 0.45], P < 0.00001, I^2^ = 0%, P = 0.47) and pMDI/MDI and demonstration/training (RR = 0.09, 95% confidence interval [CI]: [0.05, 0.17], P < 0.00001, I^2^ = 92%, P = 0.0003).

Furthermore, the test for subgroup overall effect showed a significant improvement in inhalation skills after education for assessed inhaler devices and educational approach in case of any incorrect use events (RR = 0.16, 95% confidence interval [CI]: [0.11, 0.23], P < 0.00001, I^2^ = 79%, P < 0.003). The test for subgroup differences indicates that there is statistically significant subgroup effect (P = 0.007, I^2^ = 86.4%). Accordingly, we can state that the effect of education in case of pMDI/MDI depends on the type of educational approach. The results are shown in Fig. 5.

### Results of additional analyses

We evaluated eight outcomes using certainty of the evidence (GRADE) assessment (Table 3). This analysis showed that five outcomes have moderate certainty of the evidence: (1) Occurrence of critical errors (DPI device) after education in form of demonstration, (2) Occurrence of any incorrect use events (DPI device) after education in form of verbal TTG, (3) Occurrence of any incorrect use events (DPI device) after education in form of video TTG, (4) Occurrence of any incorrect use events (DPI device) after education in form of TTG and (5) Occurrence of any incorrect use events (DPI device) after education in form of BI. These results can be interpreted that included studies provides a good indication of the likely effect assessed in meta-analysis. Furthermore, it can be concluded that there is a moderately probable chance that the effect will be significantly different.

**Table 3:** Summary of findings (SoF) of meta-analysis using Working Group Grades of Evidence (GRADE).

Outcomes	Absolute Effect		Relative effect (95% CI)	Number of studies	Certainty of the evidence (GRADE)^a^
	Without education	With education			
Occurrence of critical errors (DPI device) after education in form of demonstration	59 per 226	15 per 226	RR 0.28 [0.17, 0.47]	1 study dividend into 4	⊕⊕⊕⊖ Moderate^b^
	**Difference: 44 less errors** 0.19 (95% CI: 0.26 to 0.13 more)^a^				
Occurrence of any incorrect use events (DPI device) after education in form of verbal TTG	18 per 46	11 per 46	RR 0.62 [0.33, 1.16]	1 study divided into 2	⊕⊕⊕⊖ Moderate^b^
	**Difference: 7 less errors** 0.15 (95% CI: 0.34 to 0 fewer)^a^				
Occurrence of any incorrect use events (DPI device) after education in form of video TTG	26 per 52	2 per 52	RR 0.11 [0.02, 0.61]	1 study divided into 2	⊕⊕⊕⊖ Moderate^b^
	**Difference: 24 less errors** 0.46 (95% CI: 0.61 to 0.32 more)^a^				
Occurrence of any incorrect use events (DPI device) after education in form of TTG	21 per 28	3 per 28	RR 0.17 [0.03, 0.97]	2	⊕⊕⊕⊖ Moderate^c^
	**Difference: 18 less errors** 0.64 (95% CI: 0.84 to 0.45 more)^a^				
Occurrence of any incorrect use events (DPI device) after education in form of BI	24 per 28	19 per 28	RR 0.82 [0.57, 1.19]	2	⊕⊕⊕⊖ Moderate^c^
	**Difference: 5 less errors** 0.18 (95% CI: 0.39 to 0 fewer)^a^				
Occurrence of any incorrect use events (DPI device) after education in form of face to face method	76 per 321	12 per 146	RR 0.31 [0.07, 1.34]	2	⊕⊖⊖⊖ Very low^d^
	**Difference: 64 less errors** 0.15 (95% CI: 0.22 to 0.09 more)^a^				
Occurrence of any incorrect use events (pMDI/MDI device) after education in form of face to face method	64 per 125	16 per 111	RR 0.28 [0.17, 0.45]	2	⊕⊕⊖⊖ Low^e^
	**Difference: 48 less errors** 0.37 (95% CI: 0.48 to 0.26 more)^a^				
Occurrence of any incorrect use events (pMDI/MDI device) after education in form of demonstration/training	101 per 225	9 per 225	RR 0.09 [0.05, 0.17]	2	⊕⊖⊖⊖ Very low^f^
	**Difference: 92 less errors** 0.41 (95% CI: 0.48 to 0.34 more)^a^				

*95% CI* 95% confidence interval, *RR* risk ratio, *GRADE* working group grades of evidence.

^a^The confidence interval was calculated from the difference in the proportion of the number of errors in the inhalation technique before and after education.

Explanations.

^b^The evidence was downgraded from a high to moderate rating because of a risk of bias due unclear blinding and allocation concealment. The score was then upgraded by one due to the strong association between the included outcomes and the absence of likely plausible factors.

^c^The evidence was downgraded from a high to moderate rating because of a risk of bias due unclear random sequence generation, blinding and allocation concealment.

^d^The evidence was downgraded from a high to very low rating because of non-randomised evidence and serious inconsistency (large heterogeneity I^2^ = 83%, P value [P = 0.01] due to too many participants in one of the groups, which may distort the results.

^e^The evidence was downgraded from a high to low rating because of non-randomised evidence.

^f^The evidence was downgraded from a high to very low rating because of non-randomised evidence and unexplained serious inconsistency (large heterogeneity I^2^ = 92%, P value [P < 0.0003].

One outcome: Occurrence of any incorrect use events (pMDI/MDI device) after education in form of face to face method, showed low certainty of the evidence. This result can be interpreted as providing some indication of the likely effect. It should be emphasized that, the probability that it will be significantly different is high.

Two remaining outcomes: (1) Occurrence of any incorrect use events (DPI device) after education in form of face to face method and (2) Occurrence of any incorrect use events (pMDI/MDI device) after education in form of demonstration/training showed very low certainty of the evidence. In this case, one can interpret that these results do not provide reliable indications of the probable effect. In addition, there is a very high probability that the estimated effect will be significantly different. The above interpretation was developed based on the GRADE guidelines^28^.

Four studies included in quantitative synthesis have high risk of bias due to lack of randomization (selection bias). Additionally, one of the studies in sensitivity analysis included an age group of 15 years and older. We decided to include this study in the meta-analysis because adolescents aged 15 years and older can effectively participate in education and achieve satisfactory results, as well as young adults (18 and older). Therefore, these studies were eligible to sensitivity analysis (Table 4). In one case, the assessed findings were robust to sensitivity analysis, which showed slight differences in the results after excluding studies with a high risk of bias from the meta-analysis. In two cases, however, excluding studies from the meta-analysis resulted in the inability to perform a new comparison.

**Table 4:** Sensitivity analysis of studies included in a meta-analysis.

Outcome	Study ID	Risk Ratio [95% CI] before sensitivity analysis	Risk Ratio [95% CI] after sensitivity analysis
Occurrence of any incorrect use events errors (DPI device) after education in form of face to face method	Aydemir et al.^20^ Nitya et al.^16^	0.38 [0.21, 0.70] P = 0.002 I^2^ = 81% (P < 0.00001) Test for subgroup differences: Chi^2^ = 8.94, df = 4 (P = 0.06), I^2^ = 55.3%	0.41 [0.20, 0.81] P = 0.01 I² = 80% (P < 0.0001) Test for subgroup differences: Chi² = 7.88, df = 3 (P = 0.05), I² = 61.9%
Occurrence of any incorrect use events (pMDI/MDI device) after education in form of face to face method	Aydemir et al.^20^ Nitya et al.^16^	0.16 [0.11, 0.23] (P < 0.00001) I^2^ = 79% (P = 0.003) Test for subgroup differences: Chi^2^ = 7.34, df = 1 (P = 0.007), I^2^ = 86.4%	Not applicable
Occurrence of any incorrect use events (pMDI/MDI device) after education in form of demonstration/training	Aksu et al.^30^ Balamurugan et al.^31^		Not applicable

*DPI* dry powder inhaler, *pMDI* pressurized metered dose inhaler.

## Discussion

To date, few systematic reviews and meta-analyses on proper inhalation technique have been published. The issues related to the frequency of errors rate made by patients while using the inhalation device were raised^36^. Also the prevalence and types of device errors^37,38^ and patients’ preferences regarding inhaler features (including size, medication administration, durability, a dose counter, portability, perceived ease of use and dose preparation) were assessed. Systematic reviews assessed the clinical effectiveness and cost-effectiveness of inhalers are also available^39^. Furthermore, an attempt was made to assess clinical outcomes and exacerbation rates after provided educational program in older adults^40^. Meta-analysis data on the impact of pharmacist-delivered education on asthma clinical outcomes, quality of life, and medication adherence were presented^41,42^.

After review of the literature, we can conclude that issues regarding patient education in systematic reviews and meta-analyses could be continued in further studies because they have not been fully explored. Our meta-analysis brings a new perspective by approaching the issue in the context of the influence of the type of educational approach on the effect of education. Our study included a different methodology by comparing the effect before education to the effect after education regarding the occurrence of any incorrect use events and critical errors, which was not considered in the previously mentioned meta-analyses. This concept makes this study novel but, at the same time, complementary to already published meta-analyses.

Among factors affecting compliance with inhalation therapy, one of the most important is educating patients on the correct inhalation technique^43^. Inappropriate inhalation observed in patients is a well-known problem but has remained unsolved for many years^16,43^. A factor affecting the ability to use an inhaler properly may be that patients use different inhalers simultaneously, which differ in the required inhalation technique. Additionally, approximately 30% of these patients have never been educated in this area^43^. Many different types of inhalers are available and are supposed to be accessible to the patient. However, as we showed in our meta-analysis, most patients made mistakes when using them before education.

Dabrowska et al.^43^ showed that while a single training session has been shown to reduce errors in the inhalation technique, its effects are not long-lasting. This means that the effect of a single inhalation training is temporary. It was observed that the effect of inhalation training decreased 6 months after the education. The temporary nature of these effects underscores the need for continuous patient education. It has been demonstrated that the benefits of a single training session diminish over time and should be reinforced.

It is also important to consider the time needed to improve the use of inhalation devices and how long the education lasted. Melani et al.^44^, considering that repeated education is required to ensure the persistence of good inhaler use, investigated whether the time required to achieve the correct inhaler technique differed between devices. In this study, the authors reported that the mean education time required to correct inhaler misuse was shorter for DPIs than for MDIs. After selecting studies for the systematic review and meta-analysis, we noticed that although Melani et al.^44^ raised important conclusions almost a decade ago about differences in the time required to achieve the correct inhaler technique between devices, many studies fail to mention this factor. Among the studies in our systematic review, only two^8,32^ consider the time needed to achieve the correct inhalation technique (Supplementary Table 2).

This indicates another knowledge gap, as new studies lack information on an important factor in assessing the effectiveness of education - time. Although our meta-analysis was not designed to assess the time required to achieve the correct inhaler technique, as this was not included in the research question (PICO), after noticing the lack of information regarding the time required to achieve satisfactory educational outcomes and its duration, we decided to briefly discuss this issue.

Although our research question did not include this aspect, the role of the educator should also be emphasized. There are many reports in the literature about medical personnel educating patients in the field of the inhalation technique^45^. These can be physicians, nurses, and a pharmacist^46^. Educating medical personnel is also emphasized so they can provide it correctly to the patient. Studies have shown that medical personnel often do not have the appropriate knowledge in this aspect and require training^47–49^. Our systematic review and meta-analysis showed that physicians, pharmacists, trained lung function technicians, and trained research educators. The group of education providers in the studies we analyzed is complex. However, in most cases, they have a common denominator: the prior acquisition of appropriate qualifications to impart knowledge on the correct inhaler technique. Therefore, it can be assumed that the person who provided education did not significantly influence its effect if they had prior training by the applicable standards.

An additional important aspect is the method used to assess the inhaler technique. Our systematic review showed that patients’ skills can be assessed in different ways and configurations depending on the study design. However, as we have shown in our systematic review (Supplementary Tables 1 and 2), the authors used standardized, well-known “checklists” in many of the studies we reviewed. Obviously, “checklists” may differ slightly depending on the device, even within one type of inhaler. However, the concept of operation of devices belonging to one type is the same. Therefore, any differences in checklists do not constitute a bias in comparing the effects of the type of education we conducted in the meta-analysis. When discussing issues related to the assessment of inhaler technique, it is also important to consider the different types of errors and their definitions, and the fact that they may differ between devices. This is particularly crucial for critical errors^50^. In our meta-analysis (Fig. 3), we combined data from one study for two different inhalers in a single forest plot of critical errors. The study authors defined critical errors, which we have included in the Supplementary Materials in Table 1. If a meta-analysis were to compare different studies using different devices and definitions of critical errors, the risk of bias would be very high. However, we combined the results for critical errors with the exact definition, which significantly reduces this risk.

It should also be emphasized that there are cases where an inhaler device error is not solely related to a lack of knowledge of the proper technique. This can be explained by the example of DPI and pMDI devices included in our meta-analysis. DPI require a forceful and deep inhalation. This approach allows for the dispersion of coarse particles attached to a lactose carrier^51^. An error in this area can be made by a person who does not know that they are supposed to inhale strongly and deeply and by someone who knows that they are supposed to do so but may not be able to exert a sufficient inhalation effort. This may be related to patients having varying degrees of airflow limitation, meaning they may have lower inspiratory flows compared to healthy individuals. This, in turn, may affect the distribution of active compounds in the lungs^51,52^. In the case of pMDI inhalers, one of the difficulties associated with effective drug delivery to the lungs and, consequently, with lower therapeutic effects is coordinating the activation of the device with inhalation. This is particularly difficult for small children and the elderly^52^. No education may solve the problem in such cases, and the prescriber should change the device. This case indicates that each patient should be treated individually when a physician selects an inhaler. The effectiveness of inhalation therapy depends on the correct inhalation technique and the device chosen by the physician^51^.

The studies in this meta-analysis are diverse because they include both RCTs and prospective, observational, open-label and real-life. However, this approach provides a holistic view of the available results because although RCTs are considered the most reliable, real-life studies’ role is significant in respiratory diseases^53–55^. RCTs cover a homogeneous, strictly selected, and monitored patient population. Therefore, it is difficult to relate the results obtained in RCTs to situations in real medical practice settings. However, real-life studies involving a heterogeneous group of patients who often do not follow medical recommendations and are accompanied by other diseases better reflect the nature of everyday practice^55^.

The results of our systematic review and meta-analysis, indicate that regardless of the type of device, patients make mistakes when using inhalers. Additionally, regardless of the inhaler, the occurrence of errors made by patients decreases when they receive education, which we were also able to demonstrate. It should be emphasized that in our meta-analysis we managed to demonstrate an important relationship. For pMDI/MDI devices, it was observed that the type of education impacts the number of errors made in the inhalation technique. This conclusion is based on the fact that in our meta-analysis for pMDI, each subgroup is a different type of education; if the subgroup effect is statistically significant, it indicates that, in this case, the type of education influences the result^56^. We compared two subgroups. The first subgroup included two studies on education using the face-to-face method^17,21^ in which training was conducted once as a demonstration. In the second subgroup, we included two studies^31,32^ in which education in the form of a physical demonstration was also used. The training was repeated until the correct technique was achieved. Our meta-analysis showed that better results were achieved in the second subgroup.

Similar conclusions were drawn by Sestini et al.^22^ who also observed that the type of education is important in achieving better results in inhalation technique (better results are achieved when education is provided in the form of a demonstration and when the education is repeated). However, for DPI devices, we did not demonstrate such an association.

Additionally, in the analysis for DPI in terms of critical errors, due to the limited number of selected studies for comparison, we did not introduce subgroups (the data concerned one type of device and one educational method, so there was no need to introduce a subgroup).

Although more research in this area is needed to be able to apply conclusions to practice, the result we obtained may constitute a premise for conducting further research on this issue.

It should also be added that in this meta-analysis we were only able to perform analyses for two types of inhalers because among the searched studies we did not find enough data that would meet the inclusion criteria and could be used for comparisons in the meta-analysis. The reason for limiting the number of data points for comparison in the meta-analysis was our assumptions to limit the risk of bias and systematic errors. These included not mixing groups in terms of age, educational approach type, and type of the device, and randomized and nonrandomized studies.

The studies compared in a systematic review included different types of inhalers and education approaches (Table 3). However, each type of training contributed to improving patients’ inhalation skills. Kim et al.^6^ observed that even a five-minute educational session could decrease the number of incorrectly performed inhalations. However, several included studies showed that some methods were more effective than others^7,8,10^. Important outcomes were reported by Brusselle et al.^57^ in a study described an educational method involving patient training through the MyPuff app, which improved inhalation technique and adherence when switching inhalation therapy. Ahn et al.^58^ reported that the face to face method was effective in improving inhaler technique and adherence but did not improve patient quality of life. There are important findings regarding the importance of patient education and shed light on an issue that could be explored more widely. Additionally, Chrystyn et al.^34^ compared the proportion of patients making serious errors when using three different DPI inhalers and identified the device that was the most accessible to learn. Therefore, the analysis of the collected literature allows us to conclude that based on the comparison of the number of mistakes made by patients while using the inhalation device, conclusions can be drawn as to which device is associated with a greater risk of making errors and is, therefore, less accessible and more difficult for the user to use and master the inhalation technique. Although the authors of the above studies compared inhalers and tried to select the most accessible ones for patients, the results of these studies still need to be consistent. The synthesis of these results in a systematic review and meta-analysis did not allow for a clear definition of which device is most intelligible to patients before and after education in terms of the critical and any incorrect use events errors they make. Nevertheless, the studies included in the meta-analysis provided the necessary information for analysis regarding patients’ results in the field of inhalation technique before and after education. Although we encountered difficulties in selecting this data, as studies mentioned above on inhalation devices and their operation are diverse, the main goal is often not only to assess the device or patients’ skills but also to assess the effectiveness of treatment, factors influencing compliance with therapeutic recommendations or comparison different educational approaches. Therefore, for this meta-analysis, we had to select from these data those relating to the effect of education and the number of errors made by patients during inhalation. The results from the included studies also allowed for an analysis divided into subgroups according to the type of educational approach. Our meta-analysis did not show that the type of inhaler affected the number of errors before and after education. Although these conclusions should be viewed with caution due to the risk of bias, they may have practical applications and provide a basis for further analysis if more and more consistent research emerges, as they show that practitioners should pay particular attention to education and adaptation it to the patient’s needs rather than changing inhalation devices.

The results obtained in this meta-analysis should be interpreted with caution due to limitations probably related to potential confounding factors in some of the studies, such as lack of age standardization (broad age groups), unequal sample size and significant heterogeneity within some subgroups. Attention should also be paid to the risk of bias, which may be caused by the nature of the included studies, which were prospective, real-life, observational, open-label and, in some cases, non-randomized. A limitation of this meta-analysis may be that we could not perform a meta-analysis for a single type of error (included in the device checklists, Supplementary materials Table 2). It was technically not possible because there would be no studies that could be compared with each other. The studies in the comparison must be matched by device type and type of education; otherwise, there would be a risk of confounding. In some cases, authors reported errors collectively, including as critical, overall, or any errors.

## Conclusions

We observed that properly conducted patient education in the scope of the inhalation technique is effective, and its effect should not be influenced by the type of inhaler used. Before education, most patients make mistakes when using inhalers, regardless of the device. In contrast, education significantly improves inhalation techniques for all devices. This is visible in reducing the number of any incorrect use events and critical errors. In this systematic review and meta-analysis, we did not identify an educational approach not resulting in improving patients’ inhalation skills. In addition, we showed that the effect of education in case of pMDI/MDIs depends on the type of educational approach which has not been demonstrated for DPIs. Data analyzed in our study do not show that any inhaler types caused a regression in the proper use of the inhaler after education. This means that simply providing patient education is efficacious in improving inhaler use. However, the type of device is less critical regarding the number of errors made during inhalation if the patient receives high-level education in inhalation techniques. These results could be used to supplement guidelines on the optimal use of inhaled medications by reflecting practitioners about benefits of implement appropriate education which may be more important than the type of inhaler used.

## Supplementary information

Supplementary materials

## Data Availability

All data generated or analysed during this study are included in this published article.
