# Supplementary material for: Inhalation technique-related errors after education among asthma and COPD patients using different types of inhalers – systematic review and meta-analysis
Source: NPJ Prim Care Respir Med. 2025 Mar 18;35:15. doi: 10.1038/s41533-025-00422-0 (PMC11920170; doi:10.1038/s41533-025-00422-0)
Supplement: Supplementary file 1 — Supplementary materials [file 41533_2025_422_MOESM1_ESM.docx]

**Supplementary materials**

Supplementary Table 1. Checklist of the proper inhaler use and errors from the recommended technique. The checklist applies to studies included in a systematic review and meta-analysis. The sources of the checklists presented in the table are publications and supplements to publications reported by the authors of the studies included in the systematic review and meta-analysis.

| Study ID | Inhaler type | Checklist provided/cited by the studies’ authors |
| --- | --- | --- |
| Ahn et al. 2020 [58] (included in a systematic review) | DPI^*^, pMDI^*^, SMI^*^ | *“Critical steps of inhalation technique*  *Turbuhaler:*  *Open the device correctly*  *Prime with device upright*  *Seal lips around mouthpiece during inhalation*  *Inhale forcefully or deeply*  *Breezhaler:*  *Open the device correctly*  *Place capsule in the chamber*  *Close the mouthpiece*  *Press button to pierce the capsule*  *Seal lips around mouthpiece during inhalation*  *Inhale forcefully or deeply*  *Remove capsule and check for powder residue*  *Ellipta:*  *Open the device correctly*  *Seal lips around mouthpiece during inhalation*  *Inhale forcefully or deeply*  *Diskus:*  *Open the device correctly*  *Pull the lever fully back*  *Seal lips around mouthpiece during inhalation*  *Inhale forcefully or deeply*  *Genuair:*  *Open the device correctly*  *Hold the inhaler horizontally (green button facing upwards) for priming*  *Seal lips around mouthpiece during inhalation*  *Inhale forcefully or deeply*    *Respimat:*  *Twist the base one half-turn*  *Open the device correctly*  *Seal lips around mouthpiece during inhalation*  *Synchronize actuation and inhalation*  *Inhale slowly and deeply*  *pMDI:*  *Open the device correctly*  *Shake well (suspension formulations only)*  *Keep inhaler upright*  *Seal lips around mouthpiece during inhalation*  *Synchronize actuation and inhalation Inhale slowly and deeply”*  Critical errors definition:  *“Critical errors were defined as errors seriously compromising drug delivery to the lung”* |
| Aksu et al. 2016 [31]  (included in a systematic review and meta-analysis) | pMDI/MDI, DPI^*^ | *“PMDI/MDI:*  *Not removing the cap*  *Not shaking the inhaler*  *Not exhaling before actuation*  *Not holding the inhaler in the upright position*  *More actuations for a single inhalation*  *Actuation against teeth, lips, or tongue*  *Actuation in the second half of inspiration*  *Activation after end of inhalation*  *Stopping inhalation immediately after firing*  *Forceful inhalation*  *Inhalation through nose during and after actuation*  *Absent or short (<2-3 seconds) breath-holding after inhalation*  *DPI:*  *Failure to open the device*  *Failure to insert the capsule*  *Failure to pierce the capsule*  *Incorrect dose loading*  *Keeping the inhaler inclined during loading*  *Exhaling into the device mouthpiece after loading*  *Stopping inhaling prematurely*  *Inhaling by nose*  *Not sealing lips around mouthpiece during inhalation*  *Slow and not forceful inhalation*  *Exhaling into the device mouthpiece after inhalation*  *Absent breath-holding after inhalation*  *Not controlling whether some powder drug rests in the capsule after inhalation”* |
| Al-Kharouf et al. 2023 [10]  (included in a systematic review and meta-analysis) | pMDI^*^  Respimat^*^  Turbohaler, Accuhaler, Breezhaler^*^ | *“pMDI:*  *Remove cap from inhaler and spacer*  *Shake inhaler well*  *Hold inhaler upright*  *Breath out completely away from the inhaler*  *Close lips around mouth piece*  *Activate inhaler by pressing the canister once and take a deep slow breath, no whistle sound.*  *Breath in simultaneously with activation*  *Hold breath for five seconds while removing the MDI from your mouth then breath normally*  *Breath normally for 30-60 seconds, Repeat the sequence for second inhalation*  *Accuhaler:*  *Open the accuhaler by pushing the thumb grip right around until it clicks.*  *Keep inhaler horizontally*  *Push the lever away until it stops*  *Note the decrease in the number on the dose counter*  *Breath out as much as possible away from the inhaler*  *Put the mouth piece between your lips and take a strong deep steady breath*  *Remove the accuhaler and hold breath for 10 seconds then breath out slowly*  *Close the accuhaler by sliding the thumb grip back to its original position*  *Rinse your mouth and gargle with water after taking your dose*  *Turbohaler:*  *Unscrew and remove the cover*  *Understand that dose counter moves every 20 doses*  *Keep inhaler upright*  *Twist grip at the base (twist right and left until you hear a click)*  *Breath out gently away from inhaler*  *Place mouth piece between your teeth (don’t bite), close lips and take a strong deep steady breath*  *Remove the inhaler from your mouth and hold for 10 seconds then breath out gently*  *Replace cover*  *Rinse your mouth and gargle with water after taking your dose*  *Respimat:*  *Preparation for use:*  *Press the safety catch button while firmly pulling the clear base straight off with your other hand. Be careful not to touch the piercing element*  *Insert the narrow end of your medication cartridge into the inhaler. Place the inhaler on a flat surface, and push down firmly until it clicks into place*  *Replace the clear base by aligning the notch with the safety catch button, and slide together until it clicks*  *How to prime:*  *With the cap closed, turn the clear base in the direction of the arrows on the label until it clicks (half a turn)*  *Push up on the small, circular opening tab until the cap snaps fully open*  *With the inhaler pointed toward the ground, press the dose release button. Look for a mist, repeat steps 3 & 4 until 4 visible mists have been dispensed.*  *How to Use:*  *With the cap closed, turn the clear base in the direction of the arrows on the label until it clicks (half a turn), then Open the hinged cap by pushing up on the small, circular tab until the cap snaps fully open*  *Breathe out slowly and fully. Close your lips around the mouthpiece without covering the air vents*  *Point the inhaler to the back of your throat. While taking a slow, deep breath through your mouth, press the dark gray doserelease button and continue to breathe in*  *Hold your breath for 10 seconds or for as long as comfortable*  *Breezhaler:*  *Tilt the mouthpiece to open.*  *Remove capsule from blister and place in chamber.*  *Close mouthpiece until it clicks.*  *Press side piercing buttons in once and release. (Do not shake.)*  *Breathe out gently (away from inhaler).*  *Put mouthpiece between teeth (without biting) and close lips to form good seal Breathe in rapidly and steadily, so capsule vibrates*  *Continue to breathe in as long as comfortable.*  *Hold breath for about 5 seconds, or as long as comfortable.*  *While holding breath, remove inhaler from mouth.*  *Breathe out gently (away from inhaler).*  *Open mouthpiece and remove used capsule. If more than one dose is needed, repeat all steps starting from step 3.*  *Close mouthpiece and cap”* |
| Aydemir et al. 2015 [21]  (included in a systematic review and meta-analysis) | DPI  pMDI/MDI | *“DPI:*  *Failure to remove cap Failure to insert the capsule Failure to pierce the capsule Blowing into the device before inhalation Inhalation is not forceful from the start Inhalation through the nose*  *PMDI/MDI:*  *Failure to remove the cap Not holding the inhaler upright (if the drug does not come out of the mouthpiece) Actuation not corresponding to inhalation; actuation before inhalation Actuation not corresponding to inhalation; actuation is too late Failure to actuate Failure to inhale Inhalation through the nose”* |
| Balamurugan et al. 2020 [32]  (included in a systematic review and meta-analysis) | pMDI,  Synchrobreathe^*^ | *“pMDI/MDI:*  *Shake the inhaler well*  *Remove the mouthpiece cover and check that the mouthpiece is clean*  *Hold the inhaler upright as shown, with your thumb on the base*  *Place either one or two fingers on top of the canister*  *Breathe out as far as is comfortable, through your mouth*  *Place the mouthpiece of the inhaler in your mouth between your teeth and close your lips around it (do not bite it)*  *Start breathing in slowly through your mouth*  *As you breathe in, press down the canister to release and dose while continuing to breathe steadily and deeply*  *Remove the inhaler from your mouth and hold your breath for 10 seconds or for as long as it is comfortable*  *Breathe out slowly*  *After use, replace the mouthpiece cover*  *Synchrobreathe:*  *Shake the Synhrobreathe well*  *Open the inhaler, by folding down the mouthpiece cap*  *While sitting or standing, hold the Synchrobreathe upright as shown*  *Breathe out normally as far as is comfortable, through your mouth*  *Place the mouthpiece of the Synchrobreathe in your mouth; between your teeth and close your lips around it (do not bite it)*  *Start breathing n slowly and deeply through the mouthpiece*  *The Synchrobreathe will automatically release a dose when you begin to breathe in from the mouthpiece.*  *Do not stop breathing in when you hear the click and whoosh, and feel the dose in your mouth*  *It is important to keep breathing in after the puff is released”* |
| Brusselle et al. 2023 [57]  (included in a systematic review) | pMDI/MDI^*^ | Check list not provided |
| Chrystyn et al. 2016 [34]  (included in a systematic review) | Diskus^*^  Pulmoject^*^  Turbohaler^*^  (empty devices) | *“Pulmoject:*  *Does not remove cap^**^*  *Green window (dose actuator) not checked*  *Places fingers or mouth completely over holes in the device body (restricting airflow)^**^*  *Fails to put in mouth and seal lips around mouthpiece^**^*  *Inhalation is not as fast as possible from the start (defined as audible 'soft plop' heard during the first half of inhalation)^**^*  *Cap is not securely screwed down tightly onto base (with a snug fit) and an audible click is not heard^**^*  *Failure to inhale through mouthpiece^**^*  *Inhalation through the nose at the same time as through the mouth*  *Ask patient how they know when their device is empty - Does not know*  *Diskus:*  *Does not slide cover as far as possible^**^*  *Does not slide lever fully to open mouthpiece^**^*  *Holds in a downward position after dose preparation (before an inhalation)*  *Shakes after dose preparation^**^*  *Blowing into the device before inhalation*  *Fails to put in mouth and seal lips around mouthpiece^**^*  *Inhalation is not as fast as possible from the start (defined as a very fast suck) / Inhalation is not forceful from the start^**^*  *Failure to inhale through mouthpiece^**^*  *Inhalation through the nose at the same time as through the mouth*  *Ask patient how they know when their device is empty - Does not know.*  *Turbohaler:*  *Does not remove cap^**^*  *Places fingers or mouth completely over holes in the device body (restricting airflow)*  *Fails to put in mouth and seal lips around mouthpiece^**^*  *Shakes during preparation^**^*  *Device not held upright (mouthpiece skywards) when the base is twisted during dose preparation^**^*  *Dose not prepared correctly- twisting the base until it clicks^**^*  *Dose not prepared correctly- turn it back to the original position^**^*  *Device not held upright (mouthpiece skywards) after the base is twisted until inhalation^**^*  *Shakes after dose preparation^**^*  *Exhales into the inhaler before taking dose^**^*  *Inhalation is not as fast as possible from the start (defined as a very fast suck) / Inhalation is not forceful from the start^**^*  *Failure to inhale through mouthpiece^**^*  *Inhalation through the nose at the same time as through the mouth*  *Cap is not securely screwed down tightly onto base*  *Ask patient how they know when their device is empty - Does not know”*  Critical/serious errors definition:  *“Serious inhaler technique errors, defined as errors that could affect adequate dose delivery to the lungs (also referred to as critical or major errors”* |
| Kim et al. 2021 [6] (included in a systematic review) | pMDI/MDI^*^ | *“Put the metal canister into the “boot” making certain it is seated correctly*  *Shake the inhaler several times. This mixes the propellant and medicine*  *Remove the cap off from the mouthpiece Breathe out to the end of a normal breath*  *Hold the inhaler in its upright position (with the mouthpiece at the bottom)*  *Put the mouthpiece in your mouth, past your teeth and above your tongue.*  *Close your lips around the mouthpiece so that the medication does not go in your eyes*  *While breathing in slowly and deeply through your mouth, fully press down once on the top of the metal canister of your inhaler Hold your breath for 5 to 10 s Breathe out slowly If you take more than one spray, wait 15 to 30 s (or as directed in the package insert) before taking the next puff*  *Then repeat steps 3–9 Replace the cap on the mouthpiece after you are finished*  *If you are inhaling a steroid, rinse your mouth out with water, swish, gargle and spit”* |
| Nitya et al. 2021 [17]  (included in a systematic review and meta-analysis) | pMDI  DPI  pMDI with spacer^*^ | *“DPI:*  *Open the inhaler mouthpiece, or remove the cap Place the capsule(s) in the inhaler and insert it into the Rotacap hole with its transparent end facing downward and rotate the base Breathe out fully, away from the inhaler Hold the inhaler as directed.*  *Do not cover the vents.*  *Place the mouthpiece between your lips Take a fast, deep, forceful breath in through your mouth Remove the inhaler Hold the breath for 10 s Remove the inhaler from the mouth and breath out slowly away from the inhaler.*  *Wait for 1 min in the case of second dose*  *pMDI/MDI:*  *Remove the cap Shake the inhaler as directed Hold the inhaler upright with the mouthpiece at the bottom with the thumb below the base and the finger on the top of the canister Tilt your head back slightly, and breathe out slowly and completely away from the mouth piece Place the mouth piece between the teeth and close the lips As you start to breathe in, press down on the inhaler one time and continue to breathe in slowly and deeply for 3‐5 s*  *Hold the breath for 10 s Remove the inhaler from the mouth and breath out slowly away from the inhaler.*  *Wait for 1 min in the case of second dose”* |
| Press et al. 2012 [8] (included in a systematic review and meta-analysis) | DPI and pMDI/MDI^*^ (no information about the spacer available, but the authors reported that they used a checklist, which, upon checking the provided source, turned out to be a checklist for MDI with a spacer). | *“DPI:*  *Uses thumb or finger in thumb grip to open device until the mouthpiece appears*  *Keeps Diskus horizontal prior to step 3 and until step 7 completed Slides lever once until it clicks Breathes out fully*  *When breathing out fully (step 4), does so away from Diskus Presses lips tightly above and below mouthpiece opening Breathes in quickly, filling lungs with medicine Holds breath for at least 5 seconds (with or without Diskus in mouth)*  *Removes Diskus before breathing normally*  *Closes Diskus by placing thumb or finger in the thumb grip and sliding it closed*  *pMDI/MDI*  *Removes cap of inhaler and spacer*  *Shakes inhaler up and down*  *Attaches inhaler to back of spacer*  *Breathes OUT fully*  *When breathing out fully (step #4), does so away from spacer/MDI*  *Puts spacer mouthpiece or MDI mouthpiece (if not using spacer) into mouth, closes lips around mouthpiece*  *Activates inhaler by pressing down on canister 1 time*  *Breathes IN SLOWLY, filling lungs with medicine. No whistle should be heard*  *Holds breath for at least 5 seconds (with or without spacer in mouth)*  *Removes spacer/MDI from mouth before breathing normally*  *Breathes normally for at least 30-60 seconds*  *Repeats sequence for second puff”* |
| Press et al. 2016 [7] (included in a systematic review and meta-analysis) | MDI (with spacer)^*^, Diskus | *“DPI:*  *Uses thumb or finger in thumb grip to open device until the mouthpiece appears*  *Keeps Diskus horizontal prior to step 3 and until step 7 completed Slides lever once until it clicks Breathes out fully*  *When breathing out fully (step 4), does so away from Diskus Presses lips tightly above and below mouthpiece opening Breathes in quickly, filling lungs with medicine Holds breath for at least 5 seconds (with or without Diskus in mouth)*  *Removes Diskus before breathing normally*  *Closes Diskus by placing thumb or finger in the thumb grip and sliding it closed*  *MDI with spacer:*  *Removes cap of inhaler and spacer*  *Shakes inhaler up and down*  *Attaches inhaler to back of spacer*  *Breathes OUT fully*  *When breathing out fully (step #4), does so away from spacer/MDI*  *Puts spacer mouthpiece or MDI mouthpiece (if not using spacer) into mouth, closes lips around mouthpiece*  *Activates inhaler by pressing down on canister 1 time*  *Breathes IN SLOWLY, filling lungs with medicine. No whistle should be heard*  *Holds breath for at least 5 seconds (with or without spacer in mouth)*  *Removes spacer/MDI from mouth before breathing normally*  *Breathes normally for at least 30-60 seconds*  *Repeats sequence for second puff”* |
| Van der Palen et al. 2013 [33]  (included in a systematic review and meta-analysis) | Diskus,  Elpenhaler | *“Diskus:*  *Preparing the dose*  *Open inhaler^**^*  *Push lever back completely^**^*  *Inhaler is kept horizontal^**^*  *Exhale to residual volume*  *Do not exhale in mouthpiece^**^*  *During inhalation mouthpiece between*  *teeth and lips^**^*  *Inhale forcefully and deeply^**^*  *Hold breath for several seconds*  *Close inhaler*  *Elpenhaler*  *Take blister strip from storage compartment^**^*  *Open protective cap^**^*  *Push back mouthpiece to reveal supporting surface^**^*  *Place blister strip correctly on supporting surface^**^*  *Close mouthpiece correctly^**^*  *Gently pull the protruding end of the strip^**^*  *Exhale to residual volume*  *Do not exhale in mouthpiece^**^*  *Mouthpiece between teeth and lips^**^*  *Inhale forcefully and deeply^**^*  *Hold breath for several seconds*  *Close inhaler^**^*  *Indicate that failure to correctly perform the specific step may lead to a crucial error and, consequently, reduced or no drug effect.”*  Critical errors definition:  *“Critical errors were defined as those that will lead to an insufficient drug deposition in the lungs.”* |

DPI: dry powder inhaler; pMDI: pressurized metered dose inhaler; SMI: soft mist inhaler;

^*^ Results not included in the meta-analysis due to lack of reference group.

*^**^ Definitely serious errors/critical errors.*

Supplementary Table 2. Additional characteristics and methodological aspects of included studies for selected attributes.

The checklist applies to studies included in the systematic review and meta-analysis.

| Attribute | Characteristics and methodological aspects |
| --- | --- |
| Study ID  Inhaler Type  Device name  Education type  Who provided education  Methods of checking  Education time  Time to achieve improvement in inhaler use  Errors type | Ahn et al. 2020 [58]  (included in a systematic review)  pMDI^*^ (without spacer), DPI^*^, SMI^*^    DPI:Turbuhaler, Breezhaler, Genuair, Diskus, Ellipta  SMI: Respimat and pMDI (no device name)  Face to face training using the “teach-back” technique  In **“teach-back”**educational approach patients are required to explain or demonstrate their skills back after training by qualified personnel*.*  Visit 1: Training in the form of: assessment of technique before education, education in the form of **demonstration** by the nurse, then the **patient explains or demonstrates** his skills after the training. The patient is **trained until** the patient fully **understands** the inhaler and fully explains its operation.  Visit 2: **Re-evaluation** of inhalation technique, **re-training** if the patient has made a mistake.  Nurse specializing in inhaler education  Checklist  Not considered  Not considered  Critical errors |
| Study ID  Inhaler Type  Device name  Education type  Who provided education  Methods of checking  Education time  Time to achieve improvement in inhaler use  Errors type | Aksu et al. 2016 [31]  (included in a systematic review and meta-analysis, in meta-analysis data for pMDI/MDI)  pMDI/MDI (no information about spacer available), DPI^*^    Handihaler, Aerolizer, Diskus, Sanohaler, Turbuhaler  Educational method defined by Aksu et al. as a “training”  Visit 1: Initial assessment of inhaler technique using the patients’ own devices. Training in the form of a physician-led **demonstration** using the patients’ inhalers. The demonstration continued **until proper technique was achieved.**  Visit 2: After 3 months. Reassessment of inhalation using the patients’ own devices.  Physician  Checklist  Not considered  Not considered  Incorrect inhaler technique (error defined in a meta-analysis as any incorrect use event) |
| Study ID  Inhaler Type  Device name  Education type  Who provided education  Methods of checking  Education time  Time to achieve improvement in inhaler use  Errors type | Al-Kharouf et al. 2023 [10]  (included in a systematic review and meta-analysis, in meta-analysis data for Turbuhaler and Accuhaler)  DPI, pMDI (no information about spacer available)  pMDI^*^, Respimat^*^, Turbohaler, Accuhaler, Breezhaler^*^  **Verbal** teach-to-goal, **video** teach-to-goal (TTG)  Teach-to-Goal (TTG) education consists of **multiple training sessions** conducted until patients achieve their learning goals.  Educational videos were prepared that showed all the steps necessary to master the inhalation technique. The educational materials were prepared based on standard checklists used to assess inhalation technique. The videos were shared with patients via smartphone. If the patient performed a step incorrectly, the video was shown again until the technique was mastered. Patients then received a copy of the videos via What-sApp1 and were encouraged to watch the videos whenever necessary.  The TTG educational method consisted of cycles of **verbal demonstration** and assessment of inhalation technique. The assessment was conducted according to an appropriate standardized checklist.  A clinical pharmacist (research assistant)  Checklist  Not considered  Not considered  Inappropriate inhaler technique (error defined in a meta-analysis as any incorrect use event) |
| Study ID  Inhaler Type  Device name  Education type  Who provided education  Methods of checking  Education time  Time to achieve improvement in inhaler use  Errors type | Aydemir et al. 2015 [21]  (included in a systematic review and meta-analysis, in mate-analysis data for DPI and pMDI)  DPI, pMDI (no information about spacer available)  pMDI, Diskus, Turbuhaler, DPI, Easyhaler  Although the authors provided the names of the devices and separate checklists, they presented the results marked as DPI and pMDI collectively.  Face to face training  Visit 1: Inhalation technique assessment before pMDI education. The face to face training used was based on the researcher **demonstrating** the correct use of the inhaler. The training sessions lasted at least 3 minutes, using demonstration devices. The patients' inhalation technique was observed and assessed immediately after the education.  Visit 2: After one month, **re-assess the patients’ skills** in using inhalation devices.  Investigator who is a chest diseases specialist  Checklist  No considered  No considered  Incorrect inhaler technique (error defined in a meta-analysis as any incorrect use event) |
| Study ID  Inhaler Type  Device name  Education type  Who provided education  Methods of checking  Education time  Time to achieve improvement in inhaler use  Errors type | Balamurugan et al. 2020 [32]  (included in a systematic review and meta-analysis, in meta-analysis data for pMDI)    pMDI/MDI (no information about spacer available), BAI Synchrobreathe^*^  Synchrobreathe, pMDI  Educational method described by Balamurugan et al. as a “training”  Visit 1: Inhalation technique assessment before pMDI education. Patients then **received training**. **The time and number of training sessions required to perform the inhalation technique correctly were recorded.**  Inhalation technique assessment for Synchrobreathe after provision of the **patient information leaflet** (PIL). Patients demonstrated how to use the device. The number of errors made was assessed. Participants then received one-time training on how to use the device.  Visit 2: After 14 days, patients **demonstrated** the inhalation technique only once, **without training**, using both devices, and the number of errors was recorded. If the patient made errors, training in both inhalation techniques was provided again **until accuracy was achieved**. The **time** and number of **training** sessions required to perform the inhalation technique correctly were recorded for each device.  Not defined  Checklist  Not considered  Synchrobreathe required a shorter average education time to correctly perform the inhalation technique and a lower total number of attempts.  Any error (error defined in a meta-analysis as any incorrect use event) |
| Study ID  Inhaler Type  Device name  Education type  Who provided education  Methods of checking  Education time  Time to achieve improvement in inhaler use  Errors type | Brusselle et al. 2023 [57]  (included in a systematic review)  pMDI^*^ (no information about spacer available)  Trimbow  Educational method described by Bruselle et al. as a “training”  Education was provided to patients at each visit by a healthcare professional via the **MyPuff® app and/or leaflets** regarding the correct use of inhalers.  Health care professional (HCP)  The HCP observed inhalation technique and scored the inhalation technique according to the Aerosol Drug Management Improvement Team (ADMIT).  No considered  No considered  Any device error, critical device error |
| Study ID  Inhaler Type  Device name  Education type  Who provided education  Methods of checking  Education time  Time to achieve improvement in inhaler use  Errors type | Chrystyn et al. 2016 [34]  (included in a systematic review)  DPI^*^  Diskus, Pulmoject, Turbuhaler  Education in form of patient education leaflet and training video  Patients were given a patient **information leaflet**. They then performed inhalation using empty devices. In case of serious errors, a **training video** was provided, and patients repeated the inhalation.  Qualified nurse  The nurse **identified serious errors** in the device operation technique and **measured the inhalation profile** (technological assessment of the inhalation maneuver).  Not considered  Not considered  Serious errors, no serious errors |
| Study ID  Inhaler Type  Device name  Education type  Who provided education  Methods of checking  Education time  Time to achieve improvement in inhaler use  Errors type | Kim et al. 2021 [6]  (included in a systematic review)  pMDI^*^ (no information about spacer available)  Not defined  One-on-one coaching training  Assessment of inhalation technique before training. Educational training **(physical demonstration)** was conducted for approximately 5 minutes with each participant. After training, each patient was asked to repeat the inhalation technique they had learned. If any of the steps were missed or performed incorrectly, the **patient was instructed until they were able to repeat the correct steps.**  Research members trained by a by a Pulmonary & Critical Care physician  Checklist  Not considered  Not considered  Error or correct |
| Study ID  Inhaler Type  Device name  Education type  Who provided education  Methods of checking  Education time  Time to achieve improvement in inhaler use  Errors type | Nitya et al. 2021 [17]  (included in a systematic review and meta-analysis, in meta-analysis data for DPI and pMDI/MDI without spacer)  DPI, pMDI/MDI, MDI with spacer^*^  Not reported  Face to face training  Visit 1: Assessment of inhalation technique before training.  All patients who participated in the education were trained by the same researcher (**physical demonstration**). The following day, participants were asked to demonstrate the procedure they had learned during the training. Inhalation technique was assessed by the researcher using a checklist.  Visit 2: The following day, the inhalation technique was re-evaluated (post training score).  Investigator  Checklist  Not considered  Not considered  Incorrect use (error defined in a meta-analysis as any incorrect use event) |
| Study ID  Inhaler Type  Device name  Education type  Who provided education  Methods of checking  Education time (duration)  Time to achieve improvement in inhaler use  Errors type | Press et al. 2012 [8]  (included in a systematic review and meta-analysis, in meta-analysis data for DPI-Diskus)  DPI, MDI^*^ (no information about the spacer available, but the authors reported that they used a checklist, which, upon checking the provided source, turned out to be a checklist for MDI with a spacer).  Diskus  Teach-to-goal (TTG), brief intervention (BI)  TTG:  The education provided consisted **of repeated rounds of assessment and education** (maximum two rounds) to ensure participants mastered inhalation technique. A trained research educator **demonstrated proper inhalation technique**. At the end of the educational intervention, the educator conducted a final assessment of participants’ technique.  BI:  A trained research educator **read each step aloud without any demonstration.** Patients were also given a copy of the written instructions. After the education was completed, the educator conducted a final assessment of the participants' inhalation technique.  Trained research educator  Checklist  Not considered  Achieving correct inhalation technique within TTG education took three times longer than within BI.  Incorrect inhaler use (error defined in a meta-analysis as any incorrect use event) |
| Study ID  Inhaler Type  Device name  Education type  Who provided education  Methods of checking  Education time  Time to achieve improvement in inhaler use  Errors type | Press et al. 2016 [7]  (included in a systematic review and meta-analysis, in meta-analysis data for DPI-Diskus)  MDI with spacer^*^, DPI  Authors provided device name only for DPI: Diskus  Teach-to-goal (TTG) or brief instruction (BI)  Press et al. reported that descriptions of TTG and BI were as in previously mentioned study Press et al. 2012.  Trained research educators  Check list  Not considered  Not considered  Incorrect inhaler technique/misusing (error defined in a meta-analysis as any incorrect use event) |
| Study ID  Inhaler Type  Device name  Education type  Who provided education  Methods of checking  Education time  Time to achieve improvement in inhaler use  Errors type | Van der Palen et al. 2013 [33]  (included in a systematic review and meta-analysis)  DPI  Diskus, Elpenhaler  Education in form of demonstration  Van der Palen description of “demonstration”:  If an error was noted during the observation of the participants’ inhalation technique, the educator **demonstrated** the correct use of the device. The participant was then asked to **demonstrate the inhalation again and the errors were again recorded.** If the patient made another error, the educator **repeated the inhalation process step by step with the participant**. In the next step, the patient was asked to demonstrate the use of the inhaler a **third time and the errors were again recorded**. If the participant still made one or more errors, the educator discussed these errors with the patient. The patient was then asked to **repeat the maneuver again.**  Trained lung function technicians  Checklist  Not considered  Not considered  Critical errors (data for meta-analysis) and non-critical errors |

DPI: dry powder inhaler; pMDI: pressurized metered dose inhaler; SMI: soft mist inhaler; COPD: Chronic obstructive pulmonary disease; BI: brief intervention; TTG: teach-to-goal; HCP: health care professional

^*^ Results not included in the meta-analysis due to lack of reference group.
